# The role of brain-localized gamma and alpha oscillations in inattentional deafness: implications for understanding human attention

**DOI:** 10.3389/fnhum.2023.1168108

**Published:** 2023-05-25

**Authors:** Daniel E. Callan, Takashi Fukada, Frédéric Dehais, Shin Ishii

**Affiliations:** ^1^Brain Information Communication Research Laboratory, Advanced Telecommunications Research Institute International, Kyoto, Japan; ^2^Institut Supérieur de l'Aéronautique et de l'Espace, University of Toulouse, Toulouse, France; ^3^Graduate School of Informatics, Kyoto University, Kyoto, Japan

**Keywords:** inattentional deafness, EEG, gamma, alpha, natural cognition, neuroergonomics, attention

## Abstract

**Introduction:**

The processes involved in how the attention system selectively focuses on perceptual and motor aspects related to a specific task, while suppressing features of other tasks and/or objects in the environment, are of considerable interest for cognitive neuroscience. The goal of this experiment was to investigate neural processes involved in selective attention and performance under multi-task situations. Several studies have suggested that attention-related gamma-band activity facilitates processing in task-specific modalities, while alpha-band activity inhibits processing in non-task-related modalities. However, investigations into the phenomenon of inattentional deafness/blindness (inability to observe stimuli in non-dominant task when primary task is demanding) have yet to observe gamma-band activity.

**Methods:**

This EEG experiment utilizes an engaging whole-body perceptual motor task while carrying out a secondary auditory detection task to investigate neural correlates of inattentional deafness in natural immersive high workload conditions. Differences between hits and misses on the auditory detection task in the gamma (30–50 Hz) and alpha frequency (8–12 Hz) range were carried out at the cortical source level using LORETA.

**Results:**

Participant auditory task performance correlated with an increase in gamma-band activity for hits over misses pre- and post-stimulus in left auditory processing regions. Alpha-band activity was greater for misses relative to hits in right auditory processing regions pre- and post-stimulus onset. These results are consistent with the facilitatory/inhibitory role of gamma/alpha-band activity for neural processing. Additional gamma- and alpha-band activity was found in frontal and parietal brain regions which are thought to reflect various attentional monitoring, selection, and switching processes.

**Discussion:**

The results of this study help to elucidate the role of gamma and alpha frequency bands in frontal and modality-specific regions involved with selective attention in multi-task immersive situations.

## Introduction

When carrying out multiple tasks at the same time, it is often the case that performance on one or multiple tasks may degrade. This is especially true during scenarios with high task demands. Indeed, attentional mechanisms are implemented to selectively enhance relevant neural processes based on current behavioral goals. Attentional selection has been proposed to be necessary because of the limited processing capacity of the brain (Buschman and Kastner, 2015) or alternatively as a means of supporting potential action (Allport, 1987; Neumann, 1987; Edelman, 1989). For example, one may not hear someone speaking to them when they are immersed in some tasks that requires considerable attention (Cherry, 1953), such as operating a vehicle, using a smartphone, and/or playing video games. There have been several studies that have investigated the phenomenon of “inattentional deafness”, which is the inability to consciously perceive and respond to audible sounds resulting from attention being directed elsewhere (Macdonald and Lavie, 2011; Dalton and Fraenkel, 2012; Koreimann et al., 2014; Raveh and Lavie, 2015; Kreitz et al., 2016; Scheer et al., 2018). The extent to which “inattentional deafness” occurs is thought to be dependent on processes related to selective and divided attention (Lavie, 2005; Callan et al., 2018; Dehais et al., 2019a,b). The goal of this study was to elucidate brain-localized neural correlates that predict successful and unsuccessful auditory perception during a dual-task situation both before and after presentation of the auditory stimulus. Our objective was also to further identify brain-localized neural correlates that predict why some individuals perform better than others.

Cortical oscillations especially, in the gamma (>30 Hz) and alpha frequency range (8 to 14 Hz), are thought to play a large role in the underlying brain processes mediating attention (Clayton et al., 2015). It has been proposed that gamma-band activity promotes task-relevant activity in modality relevant perceptual processing regions (Clayton et al., 2015). Greater gamma-band activity means greater facilitation for attended stimuli (Golumbic et al., 2013), whereas alpha-band activity is proposed to be involved with inhibition of task-irrelevant processes (Clayton et al., 2015). Greater alpha activity usually occurs in the non-dominant task modality brain processing regions and is thought to be suppressive in nature. There have been several EEG and MEG studies that have identified source-localized activity that is related to attention and performance (Fries et al., 2001; Ergenoglu et al., 2004; Hanslmayr et al., 2007; Womelsdorf and Fries, 2007; van Dijk et al., 2008; Tallon-Baudry, 2009; Wyart and Tallon-Baudry, 2009; Rieder et al., 2011; Clayton et al., 2015; Yuan et al., 2016; Wittenberg et al., 2018; Zhou et al., 2021). Most of these studies involved visual rather than auditory processing tasks. Greater post-stimulus gamma (and to a lesser extent pre-stimulus gamma) is associated with facilitation of modality-specific attention and performance (see Rieder et al., 2011 for review; Wyart and Tallon-Baudry, 2009; Yuan et al., 2016), whereas greater pre- and post-stimulus alpha activity in modality-specific brain regions is associated with degraded attention and performance (van Dijk et al., 2008; Clayton et al., 2015). Conversely, reduction in pre- and post-stimulus alpha activity in modality-specific brain regions is associated with facilitation of attention and performance (Ergenoglu et al., 2004; Hanslmayr et al., 2007; van Dijk et al., 2008; Zhou et al., 2021).

There have been a number of studies that have investigated neural correlates of inattentional deafness (Giraudet et al., 2015; Molloy et al., 2015; Durantin et al., 2017; Callan et al., 2018; Scheer et al., 2018; Dehais et al., 2019a,b; Schlossmacher et al., 2021; Somon et al., 2022). Brain imaging studies using fMRI have identified various brain regions and networks involved with attention under dual- and multi-task situations (Dux et al., 2006; Tombu et al., 2011; Szameitat et al., 2016). One region that has been cited as being active during situations of attentional overload, often referred to as attentional bottleneck, is the superior medial frontal cortex including pre-supplementary motor area (pre-SMA). This region has been implicated with processes related to inattentional deafness (Durantin et al., 2017). The results of this fMRI study indicate that the right inferior frontal gyrus (IFG) becomes active during episodes of inattentional deafness and further reveal the presence of suppressive connectivity between this region and auditory processing regions in the right superior temporal gyrus (Durantin et al., 2017). There have also been a considerable number of electroencephalography EEG and magnetoencephalography MEG studies that have found significant differences in various event-related potentials associated with inattentional deafness (Giraudet et al., 2015; Molloy et al., 2015; Dehais et al., 2019a,b; Schlossmacher et al., 2021). Molloy et al. (2015) conducted a MEG study in which participants performed a visual search task as the dominant task. The study revealed that auditory event-related responses, which were localized in the auditory processing brain regions (including the superior temporal sulcus and posterior middle temporal gyrus), were significantly greater when the concurrent visual task had a low workload, as compared to when it had a high workload. In addition, the P300 potential, thought to reflect conscious awareness, was only present for the auditory event-related responses during the low visual workload search task. The visual event-related potentials, localized to visual processing brain regions, were shown to be differentially greater when the visual task was of high workload compared to low workload (Molloy et al., 2015). These results are consistent with facilitation of brain regions involved with the task dominant modality and inhibition of brain regions involved with the task non-dominant modality. As of yet, there have been no studies that have reported source-localized brain activity related to pre- and post-stimulus gamma- and/or alpha-band activity related to inattentional deafness.

For the related phenomena of inattentional blindness, there have also been a considerable number of experiments investigating underlying brain activity (see Hutchinson, 2019 for review). Similar to inattentional deafness, inattentional blindness is the inability to consciously perceive a fully visible object as a result of attention being directed to another task, event, or object (Mack and Rock, 1998; Simmons and Chabris, 1999). EEG and MEG studies have identified inattentional blindness-related differences in event-related potentials localized to visual processing brain regions as well as those involved with attention processing in parietal and frontal brain regions (Schubo et al., 2001; Ruz et al., 2005; Guzzon and Casco, 2011; Pitts et al., 2012; Schelonka et al., 2017; Hutchinson, 2019). Studies (Harris et al., 2020; Hutchinson et al., 2021) have shown that pre- and post-stimulus alpha activity in parietal-occipital (visual processing) regions predicts inattentional blindness. Additional support that alpha activity may be related to inattentional blindness comes from a study showing that alpha-band transcranial alternating current stimulation tACS over occipital regions induces inattentional blindness (Hutchinson et al., 2020). As was pointed out in the review by Hutchinson (2019) and investigated by Pitts et al. (2014), there have been no studies implicating gamma-band activity for inattentional blindness. This is surprising given theories implicating gamma-band activity in task-specific facilitation (Clayton et al., 2015), as well as studies implicating it in attention and performance (see Wyart and Tallon-Baudry, 2009; Rieder et al., 2011 for review; Yuan et al., 2016).

It is unclear why gamma-band activity has not been found in previous studies in relation to inattentional blindness/deafness. One possibility is that this lack of a finding may be related to the type of tasks and stimuli used in these experiments. For the most part, the stimuli and tasks used to investigate brain activity underlying inattentional blindness/deafness are often oversimplified and not representative of real-world conditions (see Hutchinson, 2019 for review). It may be the case that these artificial tasks, employed in many of these experiments, do not engage the attention system that evolved to act in more natural situations. Indeed, if one considers the hypothesis (Allport, 1987; Edelman, 1989) that attention evolved to selectively enhance neural processes based on behavioral goals directed toward specific actions—whether executed explicitly or implicitly implied—then the “naturalness” of the task appears to play a critical role in engaging the attention system. This is because it is the choice of value-dependent action that determines which modalities to enhance and which modalities to inhibit. The appearance of “limited capacity” is a natural consequence of such a selectional value-dependent-based attentional system (Neumann, 1987; Edelman, 1989). It has further been stated that in order to understand the distributed brain processes underlying natural human behavior (“natural cognition”), it is important to investigate within the context of real-world situations (Makeig et al., 2009; Gramann et al., 2014, 2021). This is a key goal of neuroergonomics (Parasuraman and Rizzo, 2008; Dehais et al., 2020). Indeed, a relevant example of this neuroergonomic approach comes from a study (Callan et al., 2018) that involved an auditory detection task in pilots in flight. This experiment, which was conducted in a real-world environment, induced a high rate of inattentional deafness. It was reported that disruption in neural phase synchrony in theta and alpha frequency bands is associated with performance decrements resulting from inattentional deafness (Callan et al., 2018). It may be the case that running experiments under natural real-world like conditions is the only way in which one can investigate the underlying neural processes that truly engage natural human attention.

This study seeks to determine brain-localized neural oscillations in gamma and alpha frequency ranges that predict auditory perceptual performance using EEG on a more engaging “natural” dual-task paradigm to induce inattentional deafness. The primary task in the experiment was to play the Nintendo Wii Skateboard Arena game that uses the Wii Balance Board for control. The virtual skateboarding task was selected for this experiment because it represents a real-world situation that is engaging and likely to induce considerable inattentional deafness. The secondary task involved an auditory stimulus difference detection task (which was in effect a 1-back task). In this task, participants had to press a button when one of two stimuli was different from the one previously presented. This task was selected as the non-dominant task to investigate inattentional deafness, rather than a simple audio detection task, because it requires utilization of echoic sensory maintenance processes thought to require greater attentional processing demands. It is predicted that pre- and post-stimulus gamma and alpha-band activity related to auditory task performance will be in accordance with theories of the role of brain oscillations for attention (Clayton et al., 2015).

The experimental tasks and predictions are outlined as follows: The Wii game is composed of six levels in which various skateboarding skills are performed. The goal for each level is to complete the tasks given in a continuous manner in the briefest amount of time. Between each level, there is a transition period of relative non-activity in which the participant pushes a button to continue on to the next level. The auditory task occurs continuously throughout the experiment. The goal of the task is to push a button every time there is a stimulus change from the previous one (auditory stimuli are presented every 2–3 s). The primary hypotheses consist of the following: It is predicted that during the skateboarding task, performance on the auditory task will be degraded as a result of the high workload of the dual-task demands imposed, initiating the phenomena of inattentional deafness. Based on theories of brain oscillations for attention (Clayton et al., 2015) outlined above, it is predicted that gamma brain activity will be greater for auditory misses than hits in brain regions known to be involved with inattentional deafness (Durantin et al., 2017) including the superior medial frontal cortex and the inferior frontal gyrus. It is further predicted that gamma-band activity will be degraded/enhanced in auditory cortical processing regions with respect to misses and hits reflecting periods of inattentional deafness and successful dual-task attentional processing. In contrast, alpha activity, that is considered to be suppressive in nature (Clayton et al., 2015), is predicted to be greater in auditory processing regions for misses over that of hits. Conversely, alpha activity is predicted to be reduced in visual and motor processing regions involved with the skateboarding task. Based on the attention literature reviewed above, and on the continuous nature of both the skateboarding and auditory tasks, we maintain that these predictions will hold for activity both pre- and post-auditory stimulus onset. To explicitly test changes in oscillatory brain activity that arise from induced and or evoked properties of the auditory stimulus that are different from ongoing processes that are present before stimulus onset, an event-related spectral perturbation ERSP analysis was conducted. This analysis takes into account baseline activity prior to stimulus onset on a single trial basis. This can have a profound effect on the pattern of brain activity from analysis of post-stimulus activity without baseline removal (Basar, 1998; Makeig et al., 2004). For example, differences in brain activity that are present both pre- and post-stimulus presentation between auditory hits and misses will likely be removed if baseline correction is applied. It is predicted that brain regions involved with attentional salience (ventral attention network), including the inferior frontal gyrus known to be involved with attentional switching (Doeller et al., 2003; Perianez et al., 2004; Tamber-Rosenau et al., 2018), will show greater ERSP for hits over that of misses. Additionally, it is predicted that primary and especially secondary auditory processing regions, involved with complex processing of the acoustic features of the stimulus, will show greater ERSP for hits over misses. This pattern of differential gamma- and alpha-band activity pre- and post-stimulus onset, as well as ERSP related activity (given above), is predicted to be a signature of auditory task performance across participants. If there is a tradeoff between performing well in auditory tasks and skateboarding tasks, we can predict a negative correlation between brain activity related to auditory hits and misses. This is especially expected for the ERSP analysis, which reflects activity underlying processes related to attentional saliency and switching.

Above, we mentioned that one method used to evaluate inattentional deafness/blindness involves manipulating workload. This approach has been employed in this study to assess the phenomenon. To confirm that the observed differences in oscillatory brain activity between hits and misses are indeed related to inattentional deafness and not merely differences in perceptual performance, we compared the experimental contrasts of auditory hits relative to misses for the dual task during Wii skateboarding (high dual-task workload) with the transition period between levels of the skateboarding task (low dual-task workload). It is predicted that the same pattern of differential activity as discussed above that occur during the skateboarding task (high dual-task workload) will be maintained when contrasted with activity between hits and misses during the transition period between levels (low dual-task workload).

## Materials and methods

### Participants

This study included 14 participants (six female participants) aged 20–52 years (mean = 23.8, SE = 2.27). A modified version of the Edinburgh handedness questionnaire (that also included questions related to “hand in which chopsticks are used” and “what foot you are better at kicking with”) revealed that 13 of the participants were completely right-handed, whereas one participant used both right and left hands depending on the task. All participants reported normal hearing and normal or corrected to normal vision. All but one of the participants had prior experience playing Wii Balance Board games. Participants that did not have experience with the Wii Fit Plus Skateboard Arena game received training on a separate day from the experiment for approximately 1 to 2 h until they could consistently reach the beginner level 6. Participants that could not perform the Wii skateboard game up to level 6 were excluded from the later experiment. There were no other exclusion criteria. Originally, there were 19 participants that were recruited for the EEG experiment but five participants were excluded for reasons including the following: extremely noisy EEG data (three participants) and machine errors related to data acquisition (two participants). The experimental procedures were approved by the ATR Human Subject Review Committee (ethics approval number 158) and were carried out in accordance with the principles expressed in the WMA Declaration of Helsinki. The confidentiality rights of all participants were observed.

### Experimental tasks and procedures

**Figure 1:**
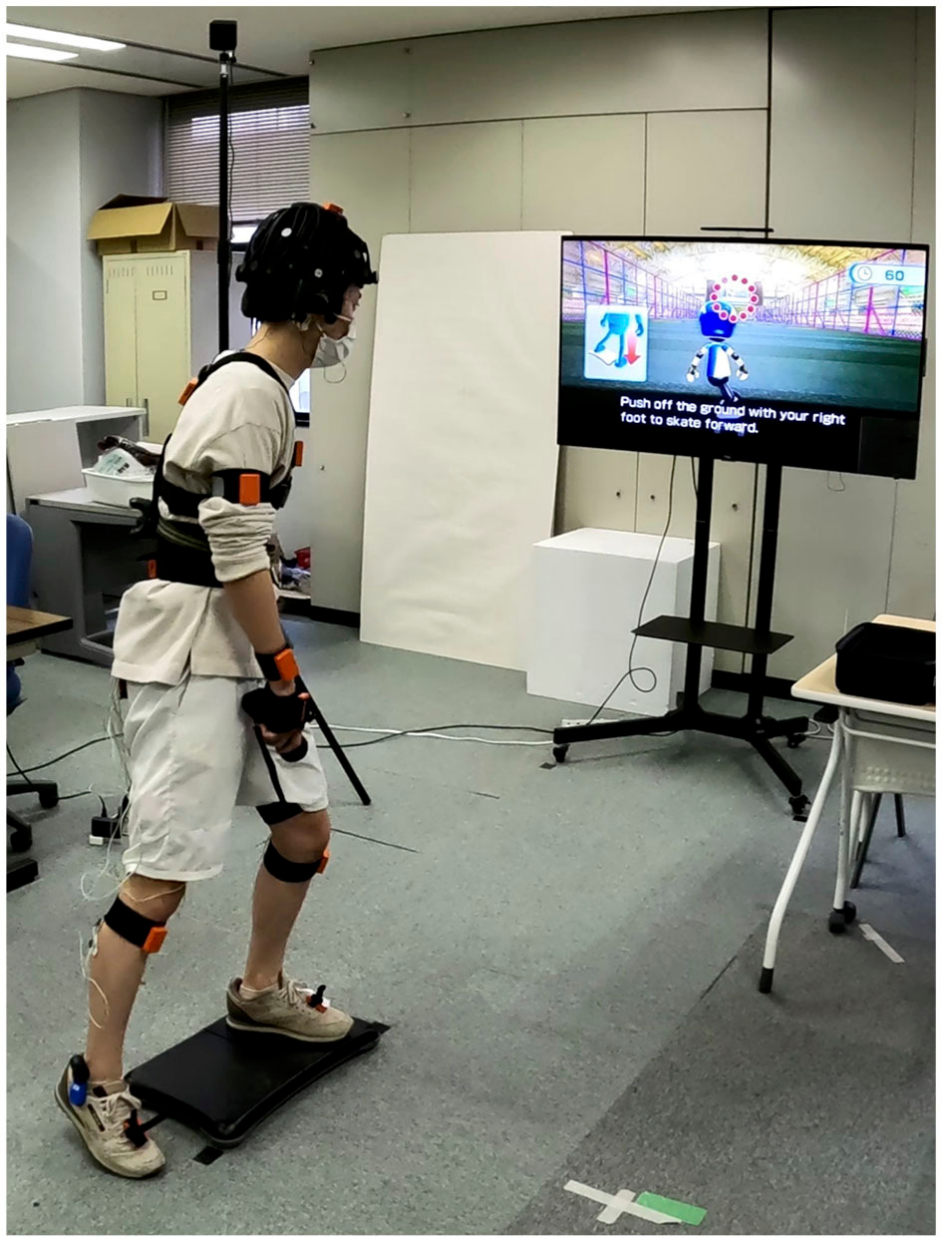
Experimental setup for dual-task virtual skateboard task and auditory difference detection task. The participant is wearing the CGX Quick-32 dry-wireless EEG and Bluetooth insert earphones. The Wii Balance Board is used to control the virtual skateboard game. Video for the skateboard game is presented on a 55-inch LCD about 2 m from the participant. Responses on the auditory task are made by pressing the B button on the Wii mote held in the right hand.

This experiment consisted of two concurrent tasks that included the following: (1) performing the Wii Skateboard Arena game; (2) performing an auditory difference detection task (see Figure 1 for a picture of the setup of the experiment with a participant doing the dual tasks). The participants were instructed to do the best they could on the Wii Skateboard Arena game by focusing their attention to it while at the same time trying to also carry out the secondary auditory difference detection task. The Wii Skateboard Arena game uses the Balance Board to detect changes in forces at the four corners of the board caused by body movement. The Wii Balance Board was placed approximately 2 m from the 55-inch LCD display upon which the video of the game was presented. No background audio sound from the game was presented. The game consists of six levels. Levels 1 to 5 focus on specific tasks [(1) maneuvering over various targets on the ground, (2) maneuvering over ramps and doing tricks, (3) doing tricks on a half-pipe, (4) jumping over and grinding on rails, and (5) jumping onto and grinding on a platform]. Level 6 is a combination of all the tasks from levels 1 to 5 with the addition of cones that must be avoided. Between each of the levels is a brief transition period in which the participant is required to press a button to proceed to the next level. Maneuvering and performing tricks in the game on the Wii Balance Board is accomplished by shifting one's weight, differentially standing on toes/heals, and by knee extensions. The participants were instructed to start the next level by pressing the A button on the hand-held Wii mote controller. At the end of each game, when level 6 was finished, a final score is displayed to the participant. The participants are instructed to restart the game after level 6 until the audio task is finished. The Wii game should be continued to be played when the audio task finishes until the end of level 6. Then, the experiment is over. The button-press responses and the Wii Balance Board force sensor data were captured by lab streaming layer (LSL, UCSD, SCCN) for synchronization with other data streams.

The auditory difference detection task required participants to push the B button on the bottom of the hand-held Wii mote controller when the current audio stimuli being played was different from the previous one played. There were two audio stimuli consisting of an upward chirp from 2 to 4 kHz (100 msec in duration) and a downward chirp from 4 to 2 kHz (100 msec in duration). A total of 400 of each type of stimuli were presented in random order with an interstimulus interval randomly determined between 2 and 3 sec. The auditory difference detection task experiment was approximately 33 min long. MATLAB was used to present the audio stimuli and send triggers to LSL identifying which stimuli are presented. The low latency EPOS GTW 270 Bluetooth earbuds were used together with a low latency aptX Bluetooth transmitter to present the audio stimuli to the participants. Two cables were routed from the audio out of the computer. One cable was to the low latency aptX Bluetooth transmitter, and the other was to the Cognionics Trigger Box. The threshold on the trigger box was set to identify the onset of the audio stimuli. The audio level was set manually for each participant prior to the experiment to be as loud as they thought they could comfortably tolerate for the 30- to 40-min experiment. The audio stimuli were played continuously throughout the experiment and therefore were played during the various Wii Skateboard Arena levels as well as between the levels. The analyses investigated event-related spectral power differences between hits relative to misses for auditory change events approximately 1 s pre-stimulus onset (−1000 to −50 msec), one half sec post-stimulus onset (0 to 500 msec), as well as an analysis in which the average spectral power from −250 to −50 msec pre-stimulus onset was used as a baseline for post-stimulus activity from 0 to 500 msec on a single trial basis [event-related spectral perturbation analyses ERSP (Makeig, 1993)]. The time segment ranges used for pre- and post-stimulus conditions were selected for the following reasons: The relatively long pre-stimulus range of approximately 1 s and the post-stimulus range of 0.5 s were selected to attempt to extract sustained attentional processes involved with suppression and enhancement related to dual-task processing thought to underlying inattentional deafness. Both the primary skateboarding task and the secondary auditory task were continuous in nature. The auditory task requires attentional maintenance of the previous stimulus in echoic memory in relation to the next stimuli that is presented every 2 to 3 s. Longer time segment ranges for pre- and post-periods were not selected to avoid potential overlap of pre- and post-brain activity. The beginning of the pre-stimulus range ensured at least 1 to 2 s of time from the previous stimulus onset. The end of the post-stimulus onset range was at least 0.5 to 1.5 s prior to the beginning of the pre-stimulus range for the next trial. The same post-stimulus time range was used for the ERSP analysis. The time period between −250 and −50 msec prior to stimulus onset was selected as a reasonable amount of time to account for baseline activity for the ERSP analysis. The reason why analyses were not carried out across multiple time points both pre- and post-stimulus onset to achieve better temporal resolution was because of the added cost of the need to correct for multiple comparisons for statistical significance.

### Physiological recording and analysis

The EEG data were measured using the Cognionics CGX Quick-32r dry-wireless EEG system (Cognionics, Inc., San Diego). The sampling rate was 500 Hz with 24-bit analog-to-digital conversion. We used 29 electroencephalography EEG channels on the headset, with ground and reference electrodes located on the temporal bone behind the left and right ear respectively. The system utilizes active electrodes to minimize external noise pickup and artifacts. In addition to EEG data, the CGX Quick-32r headset also has accelerometer data in three axes. The EEG and accelerometer data were acquired wirelessly and streamed into LSL for recording and synchronization.

The EEG data were processed using the EEGLAB toolbox (Delorme and Makeig, 2004) using a similar pipeline as given in Bigdely-Shamlo et al. (2015), Callan et al. (2018), and Sasaki et al. (2019). These preprocessing steps are used to improve signal quality and remove artifacts to be able to extract brain activity.

- The raw EEG data were band-pass filtered from 3 to 100 Hz using a Hamming windowed Sinc FIR filter.
- To extract considerable head movement artifacts from the EEG, as a result of playing the Wii Skateboard Arena game, the three accelerometer axis data in the CGX Quick-32r headset were regressed out of the EEG data (with 0 delay setting) using the CWRegrTool in EEGLAB.
- Line noise (60 Hz) was removed using the Cleanline EEGLAB toolbox (default settings).
- Automatic channel rejection was based on poor correlation to robust estimate based on other channels (0.8).
- The rejected channels were interpolated.
- Common average referencing of channels was conducted after interpolation of missing channels (An additional channel with all zeros was added so as to not lose 1 rank as a result of average referencing).
- Artifact subspace reconstruction (ASR) (see Mullen et al., 2013 and Chang et al., 2018) (Euclidian distance) was used to remove non-stationary high-variance signals from the EEG (standard deviation cutoff for removal of bursts = 20; windowed criterion = 0.25). Two analyses were conducted: one in which the time windows were removed for which ASR did not repair completely; and another in which no time windows were removed.
- Common average referencing of channels was conducted on the two datasets (time windows removed and time windows not removed) (An additional channel with all zeros was added so as to not lose 1 rank as a result of average referencing).
- ICA using PCA reduction was used on the dataset in which the time windows were removed after ASR cleaning. The number of rejected channels determined the rank reduction by PCA.
- The weights of the ICA were then applied to the ASR results without the time windows removed.
- Dipole fitting of the source for each independent component (IC) using Dipfit was conducted.
- ICLabel (version 1.1) was used to identify independent components (ICs) that are brain-related and artifact-related. ICLabel is a toolbox that allows for automated classification of ICs into seven different categories: Brain, Muscle, Eye, Heart, Line Noise, Channel Noise, and Other (Pion-Tonachini et al., 2019) at expert level of performance. ICLabel uses IC topomaps, power spectral density from 3 to 100 Hz, and equivalent current dipole to categorize each IC (Pion-Tonachini et al., 2019). The criteria of selecting “Brain” ICs in our study were based on the percentage of “brain” categorization over 50%.
- Brain-related ICs were retained, and all other ICs were removed from the dataset (only brain-related ICs were projected to the EEG electrodes).
- The Bluetooth audio presentation system latency of approximately 45 msec was corrected. The audio events were extracted from −2 s before until 2 s after presentation of the audio stimulus based on the trigger from the audio output cable.
- Source localization of the brain-related activity was carried out using LORETA Key software (Fuchs et al., 2002; Pascual-Marqui, 2002; Jurcak et al., 2007). See below for details.

### Source localization and statistical analysis

LORETA Key software employing sLORETA (Fuchs et al., 2002; Pascual-Marqui, 2002; Jurcak et al., 2007) was used to determine source localization of brain-related activity on the surface of the cortex. The position of the electrodes of the CGX Quick-Cap 32r on the head was determined by reference to the 10–10 system within the LORETA Key software. Using the LORETA Key software, the event EEG data (−2 to 2 sec) were transformed from EEG to time–frequency cross-spectrum in the gamma frequency band (30.5 to 50 Hz) and the alpha frequency band (8.5 to 12 Hz), with a window width of 250 samples (continuous Gaussian window), and a delta T between running windows equal to 25 (50msec). The time-varying cross-spectra files were then converted to sLORETA files that localizes the activity across 6,239 voxels covering the cortex with 5-mm resolution.

For each participant, the sLORETA-converted individual trials were submitted to statistical analyses using a two-sample unequal variances *t*-test for the following contrasts: Auditory change hits vs. misses for stimuli presented during levels 1 to 6 as well as during the transition period between levels. The resultant sLORETA files for these contrasts for each participant were used in random effects group level analyses using SnPM permutation analysis (5,000 randomizations) within the LORETA Key software. The random effects level analyses for data on levels 1 to 6 included single group analysis that the mean was not equal to zero as well as single group regression with the variable hit rate of levels 1 to 6 for the contrast of hits minus misses and the variable maximum Wii score across all games played during the experiment. Hit rate was used as the correlation metric rather than D-prime because the false alarm rate was low and only hit and miss trials were used in the analyses of brain activity. The maximum Wii score was selected rather than other potential metrics such as mean score as it is commonly what is used to assess who is better in video game performance. To further assess the extent to which the results can be concluded to be both a product of performance and workload (thought to underlying processes related to inattentional deafness), random effects paired analyses (using SnPM permutation analysis (5,000 randomizations) within the LORETA Key software) were carried out for these same contrasts for skateboarding levels 1 to 6 (high dual-task workload) compared to the transition period between levels (low workload). Corrected critical thresholds for multiple comparisons were used in accordance with SnPM (Nichols and Holmes, 2001). Because of our theoretical interest in the roll that the auditory cortex plays in relation to successful and impaired perception as a result of inattentional deafness, region of interest (ROI) analyses were carried out for all contrasts in both left (MNI−55,−25, 10) and right (MNI 55,−25, 10) auditory cortex based on the centroid coordinate of Brodmann area 41 and 42. Only the significant results using SnPM will be reported in the results section.

## Results

### Behavioral results

**Figure 2:**
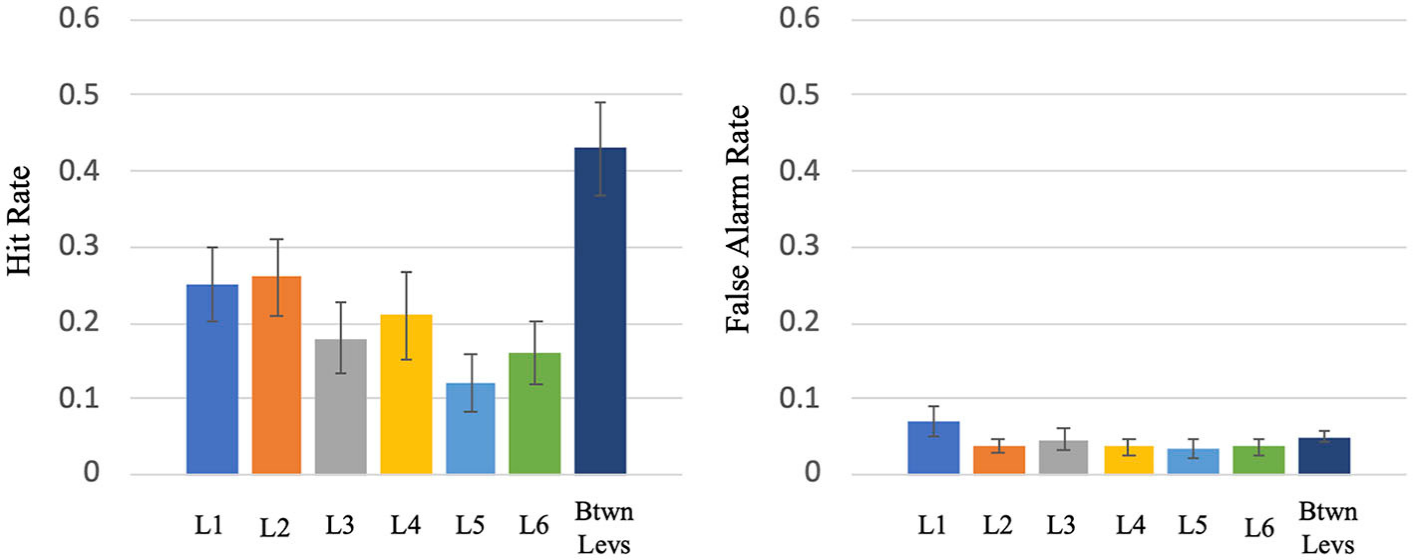
Hit rate and false alarm rate for the various levels (L1 to L6) and the transition period between levels of the virtual skateboard game.

This experiment consisted of two tasks: (1) the Wii Skateboard Arena game task; (2) the auditory difference detection task. The two tasks were done concurrently. Because a great deal of attention had to be given to the Wii Skateboard Arena task, there was low performance on the auditory difference detection task even though the stimuli were played at a high audio level and the room was quiet with no background sound from the game being presented. The mean total number of change trials across participants was 401.1 (SE = 4.2): mean hit rate = 0.30 (SE = 0.046), mean false alarm rate = 0.04 (SE = 0.009), mean d' = 1.06 (SE = 0.11). The breakdown of the mean and SE hit rates (HRs) and false alarm rates (FARs) by Wii game level segment is as follows (see Figure 2): Level 1: HR = 0.252 (SE = 0.048) FAR = 0.070 (SE = 0.020), Level 2: HR = 0.263 (SE = 0.051) FAR = 0.038 (SE = 0.010), Level 3: HR = 0.182 (SE = 0.048) FAR = 0.047 (SE = 0.016), Level 4: HR = 0.207 (SE = 0.057) FAR = 0.037 (SE = 0.011), Level 5: HR = 0.119 (SE = 0.038) FAR = 0.035 (SE = 0.012), Level 6: HR = 0.162 (SE = 0.042) FAR = 0.037 (SE = 0.011), transition period between levels: HR = 0.431 (SE = 0.061) FAR = 0.050 (SE = 0.008). For levels 1 to 6, the mean HR = 0.189 (SE = 0.044) and FAR = 0.040 (SE = 0.010). A repeated measures ANOVA test on levels 1 to 6 indicated that there is a significant difference in auditory task hit rate between the different levels, F (5,65) = 8.5, *p* < 0.001. A paired *t*-test indicated a significant difference in the mean performance of Wii levels 1 to 6 (mean HR = 0.189) compared to that of the transition period between Wii levels (mean HR = 0.431) (*T* = −7.387, *p* < 0.0001, df = 13).

There was no significant overall linear trend in Wii skateboarding game performance over the course of the experiment [Using Wilcoxon signed rank test (*p* > 0.05) that the median r score was greater than zero]. The correlation coefficient across participants ranged from *r* = 0.67 to *r* = −0.45 with the median being *r* = 0.11. The total number of games varied across participants depending on how fast they could get through the games in the same amount of time. The number of games across participants ranged from 8 to 12 with 9 being the median number of games. There were no participants that showed a significant correlation between Wii game performance and game number. Furthermore, there was no significant overall correlation between the auditory task performance (measured by hit rate) with that of Wii game performance over the course of the separate games composing the experiment [Wilcoxon signed rank test (*p* > 0.05)]. The correlation coefficient across participants ranged from *r* = 0.56 to *r* =−0.81 with the median being *r* = −0.10. There was only one participant that showed a significant correlation (*p* < 0.05, r = −0.81) between auditory task performance and Wii performance across the course of the separate games composing the experiment.

### EEG sLORETA results

#### Gamma band

**Figure 3:**
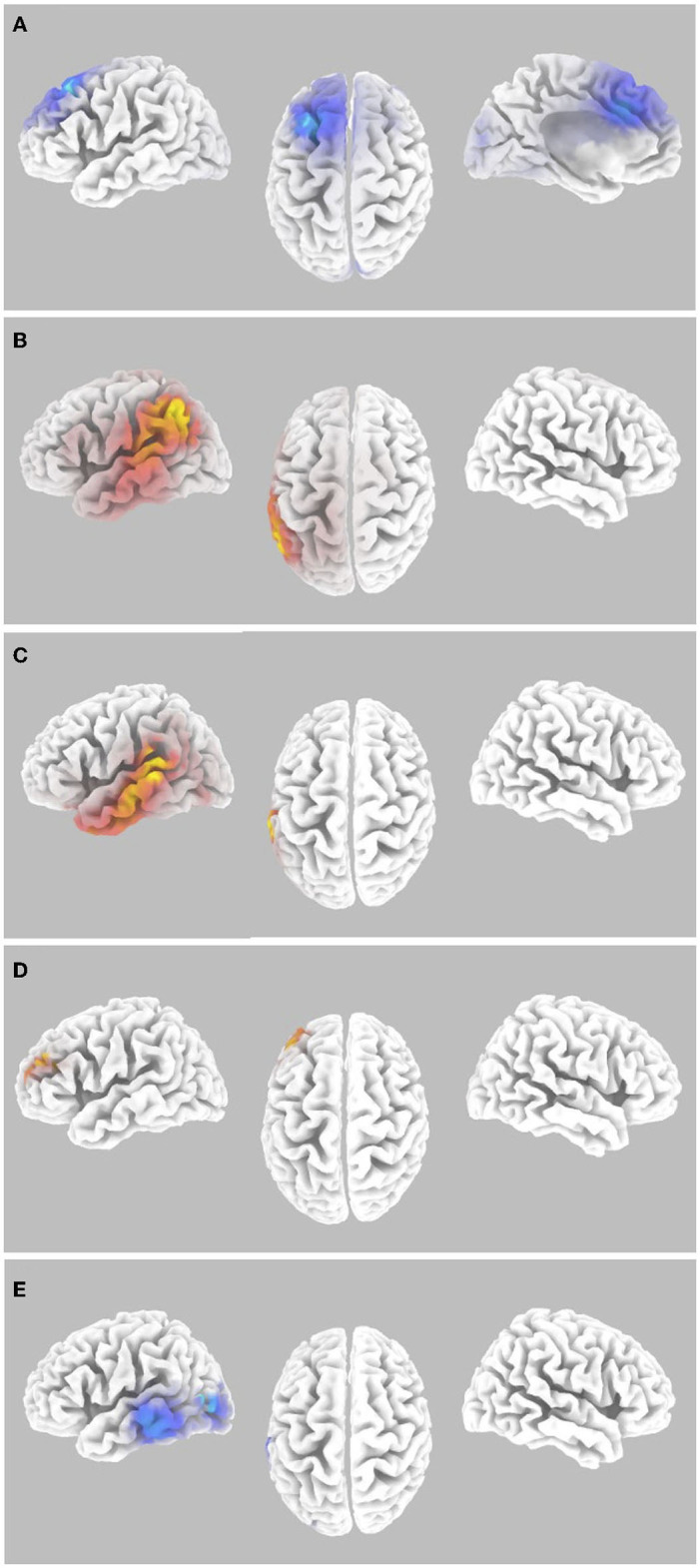
Gamma-band differences for the high workload dual-task condition occurring during the Wii skateboarding task. **(A)** Pre-stimulus onset: auditory hits relative to misses. Threshold for multiple comparisons (*p* < 0.05) rendered on the brain is *T* = −3.00 one-tail (darker blue). **(B)** Pre-stimulus onset: correlation of individual auditory hit rate with contrast of auditory hits relative to misses. Threshold for multiple comparisons (*p* < 0.05) rendered on the brain is *R* = 0.674 one-tail (red). **(C)** Post-stimulus onset: correlation of individual auditory hit rate with contrast of auditory hits relative to misses. Threshold for multiple comparisons (*p* < 0.05) rendered on the brain is *R* = 0.682 one-tail (red). **(D)** ERSP: auditory hits relative to misses. Threshold for multiple comparisons (*p* < 0.05) rendered on the brain is *T* = 4.54 1-tail (yellow). **(E)** ERSP: correlation of individual skateboard game performance with contrast of auditory hits relative to misses. No activity was found to be significant using corrected thresholds. The threshold used for displaying the figure was set to *R* = −0.57 (blue). A region of interest (ROI) analysis in auditory cortex (MNI −55, −25, 10) did reveal significant negatively correlated activity *R* = −0.655 (ROI threshold *p* < 0.05 = 0.537, two-tailed).

Random Effects level analyses were conducted for the contrast of auditory task hits vs. misses for stimuli presented during levels 1 to 6 of the Wii Skateboard Arena game. For the random effects analysis, that sLORETA gamma-band activity was not equal to zero for the contrast of hits relative to misses of average pre-stimulus power differences within the time segment from −1,000 to −50 msec, there was a significant decrease (*p* < 0.05 two-tail corrected for multiple comparisons; SnPM using 5,000 randomizations, T threshold = 3.39) in gamma-band spectral power in the superior and middle frontal gyrus (Brodmann area BA 8) extending into medial frontal gyrus (BA9) and pre-SMA (BA6), as well as the cingulate gyrus (BA32) (see Figure 3A and Table 1). Significant activity was present in the pre-SMA determined by the atlas defined by Sallet et al. (2013). No significant power differences (*p* > 0.05 one-tail corrected for multiple comparisons; SnPM using 5,000 iterations) for the contrast of hits relative to misses were present for the average of the time segment from 0 to 500 msec post-stimulus onset.

**Table 1:** Gamma band, High Workload: Hits relative to Misses.

**Contrast**	**Brain region**	**MNI coordinate x, y, z**	**RorT**
Pre	SFG BA8	−30, 25, 55	−3.48
	MFG BA8	−20, 20, 45	−3.46
	CG BA32	−10, 20,40	−3.40
	SMFC Pre-SMA BA6,8,9	−10, 25, 40	−3.39
Pre RGR HR	IPL BA40	−55, −55, 45	0.818
	AG BA39	−55, −65, 35	0.808
	SMG BA40	−60, −55, −35	0.804
	SPL BA7	−40, −60, 50	0.766
	AC BA42	−65, −35, 20	0.802
	AC BA41	−55, −30, 10	0.790
	STG BA22	−60, −40, 20	0.796
	STG BA22	−65, −25, 0	0.777
	MTG BA21	−65, −15, −10	0.768
	ITG BA20	−65, −20, −20	0.755
	Insula BA13	−45, −35, 20	0.762
Post RGR HR	AudCor BA42,22	−65, −35, 20	0.755
	STG BA22	−61, −33, 7	0.732
	MTG BA21	−60, −19, −11	0.748
ERSP	SFG, DLPFC BA10,46	−35, 45, 25	4.59

BA, Brodmann area; SFG, superior frontal gyrus; MFG, middle frontal gyrus; CG, cingulate gyrus; SMFC, superior medial frontal cortex [pre-SMA as determined by the atlas defined by Sallet et al. (2013)]; IPL, inferior parietal lobule; AG, angular gyrus; SMG, supramarginal gyrus; SPL, superior parietal lobule; AC, auditory cortex; STG, superior temporal gyrus; MTG, middle temporal gyrus; ITG, inferior temporal gyrus; SFG, superior frontal gyrus; DLPFC, dorsolateral prefrontal cortex. BA46 was determined by atlas of Sallet et al. (2013).

Random effects single group regression analyses were used to determine how participant level performance on the auditory task related to corresponding differences in brain activity. For the sLORETA contrast of hits relative to misses on the auditory task, using each individuals hit rate as the regression variable, statistically significant positive correlation (*p* < 0.05 two-tail corrected for multiple comparisons; SnPM using 5,000 iterations; R threshold = 0.742) was present for averaged pre- stimulus activity (−1,000 to −50 msec) in left inferior parietal lobule (BA40), supramarginal gyrus (BA40), angular gyrus (BA39), and auditory processing areas in the left temporal lobe including auditory cortex (BA41, 42), superior temporal gyrus (BA22), and middle temporal gyrus (BA21) (see Figure 3B and Table 1). For averaged post-stimulus activity from 0 to 500 msec, a significant positive correlation between brain activity and hit rate (*p* < 0.05 two-tail corrected for multiple comparisons; SnPM using 5,000 iterations; R threshold = 0.740) was present in left auditory processing areas including the primary auditory cortex (BA42), the superior temporal gyrus STG (BA22), and the middle temporal gyrus MTG (BA21) (see Figure 3C and Table 1).

It should be noted that there was no statistically significant correlation (*p* > 0.05 uncorrected) between brain activity (sLORETA) for hits alone and individual participant hit rate on the auditory task nor between brain activity for misses alone and individual participant hit rate. The lack of a significant correlation of brain activity, for the unitary variables of hits or misses alone, with participant performance (hit rate) strongly suggests that the significant correlation in brain activity found for the contrast of hits minus misses (Figures 3B, C and Table 1) is actually related to the difference rather than any particular variable characteristic of one of the unitary variables of hits or misses alone (e.g., number of hit or miss trials). It should also be noted that randomly selecting trials such that the number of hits and misses were equal produced vary similar results as are shown in Figures 3B, C, suggesting that the results are not due to unequal numbers of trials between hits and misses.

An ERSP analysis that takes into account trial level baseline activity just before stimulus presentation (−250 to−50 msec) was conducted to determine changes in spectral power that are a result of the auditory event. The random effects single group analysis of the sLORETA contrast for hits relative to misses for the average time segment from 0 to 500 msec post-stimulus onset showed statistically significant (*p* < 0.05 one-tail corrected for multiple comparisons; SnPM using 5,000 iterations, T threshold = 4.54) increased gamma-band power in regions along the inferior frontal junction including the left superior frontal gyrus and middle frontal gyrus (BA10), as well as the left dorsolateral prefrontal cortex DLPFC (BA46), determined using the atlas of Sallet et al. (2013) (see Figure 3D and Table 1).

The random effects single group regression analyses between participant level performance on the auditory task and the differential ERSP of hits relative to misses in the gamma frequency range did not show any statistically significant differences (*p* > 0.05 one-tail corrected for multiple comparisons; SnPM using 5,000 iterations). However, the regression analysis between participant level maximum performance on the Wii skateboard game and differential ERSP sLORETA power differences for hits relative to misses did show a significant negative correlation in left auditory processing regions using a region of interest analysis centered in the auditory cortex (BA41, 42) at MNI coordinate (−55, −25, 10) (*R* = −0.65; *p* < 0.05 two-tail, SnPM using 5,000 iterations; R threshold = 0.537) (see Figure 3E).

**Figure 4:**
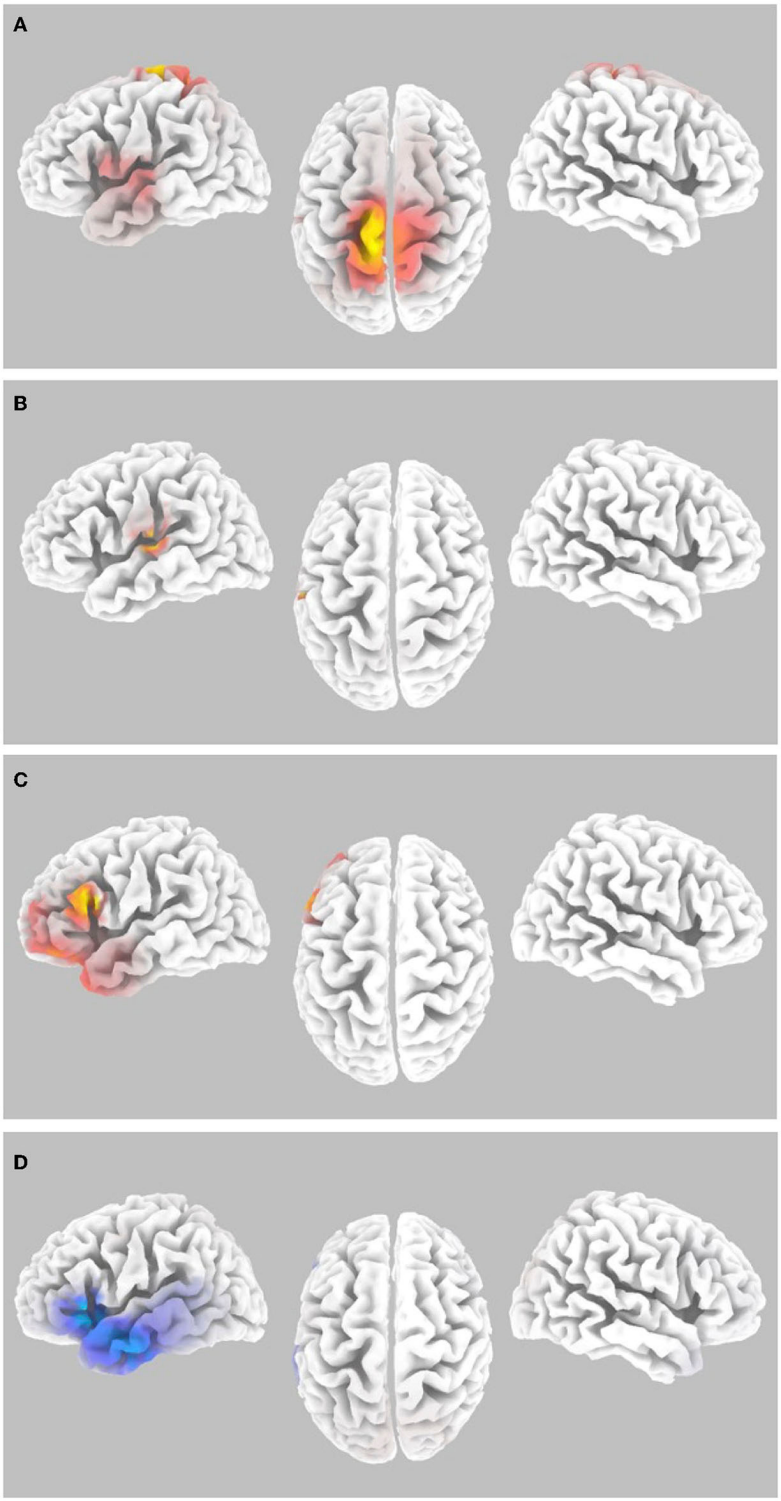
Gamma-band differences for the high workload dual-task condition occurring during the Wii skateboarding task relative to the low workload condition occurring in the transition period between Wii levels. **(A)** Pre-stimulus onset: correlation of individual auditory hit rate with contrast of auditory hits relative to misses. Threshold for multiple comparisons (*p* < 0.05) rendered on the brain is *R* = 0.712 1-tail (red). **(B)** Post-stimulus onset: correlation of individual auditory hit rate with contrast of auditory hits relative to misses. No activity was found to be significant using corrected thresholds. The threshold used for displaying the figure was set to *R* = 0.441 (blue). A region of interest ROI analysis in auditory cortex (MNI−55,−25, 10) did reveal significant negatively correlated activity *R* = – 0.467 (ROI threshold *p* < 0.05 = 0.537, one-tailed). **(C)** ERSP: auditory hits relative to misses. Threshold for multiple comparisons (*p* < 0.05) rendered on the brain is *T* = 4.31 one-tail (red). **(D)** ERSP: correlation of individual skateboard game performance with contrast of auditory hits relative to misses. Threshold for multiple comparisons (*p* < 0.05) rendered on the brain is *R* = −0.799 1-tail (blue).

To assess the contribution of both workload and auditory task performance as underlying factors of inattentional deafness for gamma-band spectral power, SnPM paired analyses of the contrasts reported above for auditory hits relative to misses during Wii skateboarding levels 1 to 6 (high workload) were compared with the same contrasts for the transition period between Wii skateboarding levels (low workload). Only the following contrasts were found to show significant differential activity. The paired group regression analyses for auditory performance for pre-stimulus brain activity showed significant differences for levels 1 to 6 relative to the period between levels in the following brain regions: precentral (BA4) and postcentral gyrus (BA3 and 5) in the most superior and medial regions corresponding to foot, leg, and trunk representation, as well as the superior temporal gyrus (BA22) (see Figure 4A and Table 2) (*p* < 0.05 one-tail corrected for multiple comparisons; SnPM using 5,000 iterations; R threshold = 0.712). The ROI analysis in left auditory cortex (MNI −55, −25, 10) was also significant (*R* = 0.643; *p* < 0.05 one-tail, SnPM using 5,000 iterations; R threshold = 0.462). For the post-stimulus contrast, no significant differential brain activity was found when correcting for multiple comparisons (see Figure 4B). However, the ROI analysis did reveal significant correlation in left auditory cortex (MNI −55, −25, 10) (*R* = 0.467; *p* < 0.05 one-tail, SnPM using 5,000 iterations; R threshold = 0.441). Both the pre- and post-stimulus analyses showed significant differential activity for the correlation with hit rate across participants in auditory cortical areas for high workload relative to low workload conditions.

**Table 2:** Gamma band, High Workload relative to Low Workload: Hits relative to Misses.

**Contrast**	**Brain region**	**MNI coordinate x, y, z**	**RorT**
Pre RGR HR	PreCG BA4	−10, −35, 70	0.758
	PreCG BA4	10, −40, 70	0.719
	PostCG BA5	−5, −45, 70	0.744
	PostCG BA3	−20, −40, 65	0.725
	STG BA22	−65, −15, 5	0.715
ERSP	IFG BA45,44	−60, 15, 20	5.46
	IFG BA47	−45, 40, −15	5.11
	MFG BA11	−35, 35, −15	5.08
	SFG BA10, 46	−35, 55, 20	4.59
	STG BA38	−35, 20, −35	4.80
	MTG BA21	−45, 10, −40	4.55
ERSP RGR Wii	IFG BA47, 45	−50, 20, 0	−0.817
	STG BA22	−50, 15, −5	−0.811

BA, Brodmann area; PreCG, precentral gyrus; PostCG, postcentral gyrus; STG, superior temporal gyrus; IFG, inferior frontal gyrus; MFG, middle frontal gyrus; SFG, superior frontal gyrus; MTG, middle temporal gyrus.

Significant differential activity for the paired group analysis for levels 1 to 6 relative to the period between levels was also present for the ERSP contrast of hits relative to misses. The brain regions found to show significant differential activity consisted of the inferior frontal gyrus IFG (BA 45,44,47), middle frontal gyrus (BA10 and 11), as well as the left dorsolateral prefrontal cortex DLPFC (BA46) and the superior temporal gyrus (BA38) (*p* < 0.05 one-tail corrected for multiple comparisons; SnPM using 5,000 iterations, T threshold = 4.306) (Figure 4C and Table 2). Activity in regions of the IFG, MTF, and DLPFC overlapped with those found in the ERSP analysis of Wii levels 1 to 6 reported above (Figure 3D and Table 1). The paired group regression analyses for Wii performance for ERSP showed significant differences for levels 1 to 6 relative to the period between levels in the following brain regions: IFG (BA47 and 45) and STG (BA22) (*p* < 0.05 one-tail corrected for multiple comparisons; SnPM using 5,000 iterations; R threshold = −0.799). The ROI analysis in left auditory cortex (MNI −55, −25, 10) was also significant (*R* = −0.513; *p* < 0.05 one-tail, SnPM using 5,000 iterations; R threshold = −0.456). Activity in the ROI analysis of the left auditory cortex was also found in the ERSP analysis of Wii levels 1 to 6 reported above (Figure 3E).

#### Alpha band

The same Random Effects level analyses were conducted for alpha band as for the gamma band above. Only the analyses reported below were significant when correcting for multiple comparisons SnPM using 5,000 randomizations. All other analyses failed to reach significance when correcting for multiple comparisons using a one-tail threshold.

**Figure 5:**
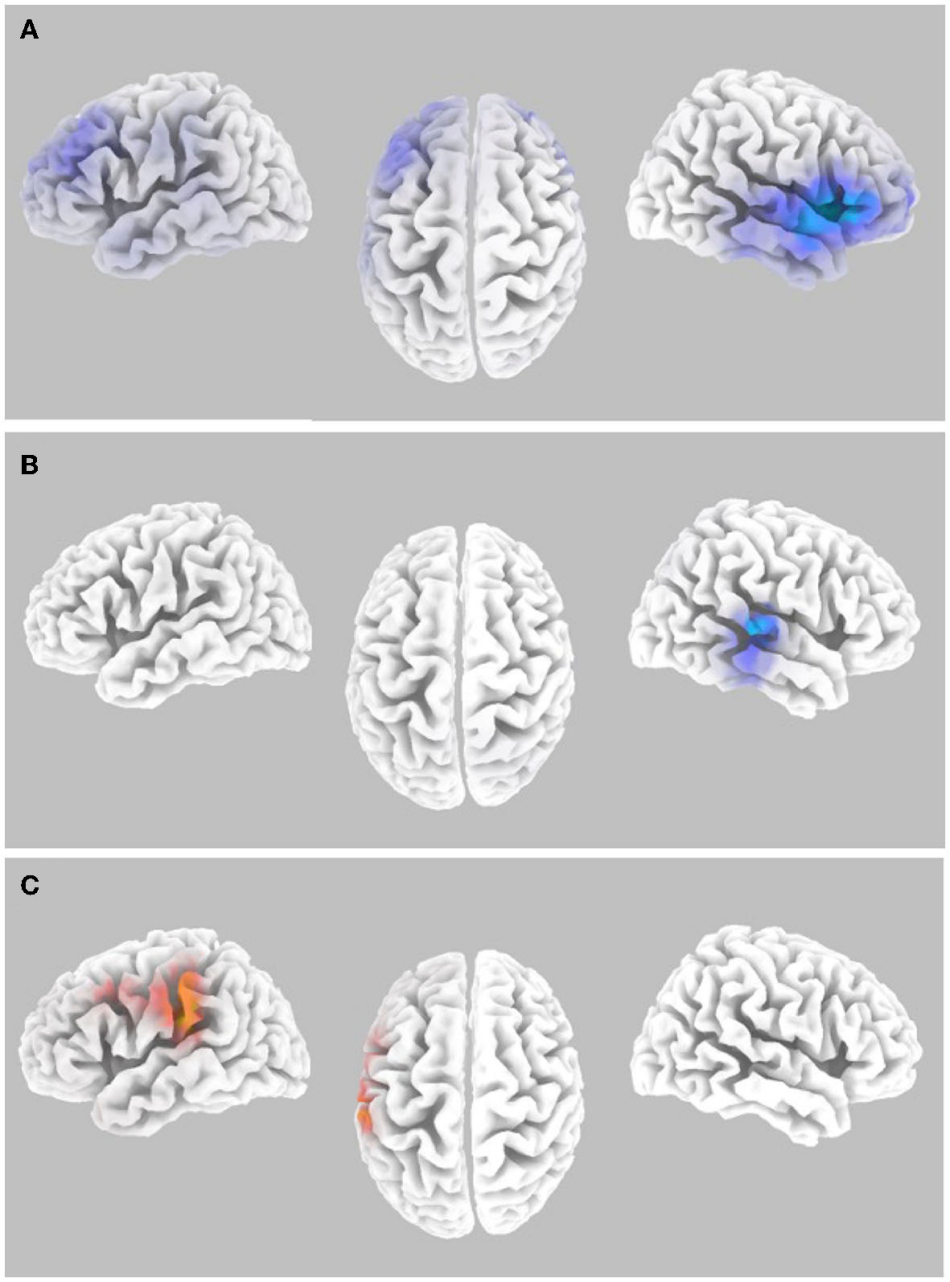
Alpha-band differences for the high workload dual-task condition occurring during the Wii skateboarding task. **(A)** Pre-stimulus onset: auditory hits relative to misses. Threshold for multiple comparisons (*p* < 0.05) rendered on the brain is *T* = 2.98 two-tail (blue). **(B)** Post-stimulus onset: auditory hits relative to misses. Threshold for multiple comparisons (*p* < 0.05) rendered on the brain is *T* = 2.86 2-tail (blue). **(C)** Pre-stimulus onset: correlation of individual auditory hit rate with contrast of auditory hits relative to misses. Threshold for multiple comparisons (*p* < 0.05) rendered on the brain is *R* = 0.696 two-tail (red).

Within the alpha frequency band, the random effects analysis over the sLORETA data for auditory task hits relative to misses of average pre-stimulus power differences within the time segment from −1,000 to −50 msec showed a significant decrease (*p* < 0.05 two-tail corrected for multiple comparisons; SnPM using 5,000 randomizations, T threshold = 2.981) in spectral power in predominantly the right insula spreading into the STG and the IFG (see Figure 5A and Table 3). The MFG and STG bilaterally also showed a significant decrease in alpha-band power (see Figure 5A and Table 3). No significant increases in alpha-band power were present when correcting for multiple comparisons (SnPM using 5,000 randomizations, one-tailed threshold).

**Table 3:** Alpha band, High Workload: Hits relative to Misses.

**Contrast**	**Brain region**	**MNI coordinate x, y, z**	**RorT**
Pre	Insula BA13	40, 10, 0	−4.15
	STG BA22	60, 0, −5	−3.99
	IFG BA47	55, 20, 5	−4.03
	MFG BA10	30, 55, −10	−3.53
	MFG BA9	−30, 25, 40	−3.56
Post	Aud Cortex BA42	65, −20, 10	−3.36
	STG BA22	65, −25, 0	−3.21
	MTG BA21	60, −30, −10	−3.02
Pre RGR HR	IPL/SMG BA40	−55, −30, 25	0.735
	PostCG BA3	−60, −25, 40	0.725
	PreCG BA4	−50, −15, 40	0.699
	PMC BA6	−50, −5, 35	0.703

BA, Brodmann area; STG, superior temporal gyrus; IFG, inferior frontal gyrus; MFG, middle frontal gyrus; MTG, middle temporal gyrus; IPL, inferior parietal lobule; SMG, supramarginal gyrus; PostCG, postcentral gyrus; PreCG, precentral gyrus; PMC, premotor cortex.

For average post-stimulus power differences (0 to 500 msec) in the alpha frequency band between hits and misses, a significant decrease was present in the right auditory cortex, STG, and MTG (*p* < 0.05 two-tail corrected for multiple comparisons; SnPM using 5,000 randomizations, T threshold = 2.863) (see Figure 5B and Table 3).

Random effects single group regression analyses were used to determine how participant level performance on the auditory change detection task (using each individual hit rate as the regression variable) related to corresponding differences in brain activity for alpha-band sLORETA hits relative to misses. For average pre-stimulus activity (−1,000 to −50 msec), a statistically significant positive correlation (*p* < 0.05 two-tail corrected for multiple comparisons; SnPM using 5,000 iterations; R threshold = 0.696) was present in the IPL extending into the somatosensory cortex in the pre- and postcentral gyrus as well as the PMC (see Figure 5C and Table 3). No negative correlations were found to be significant when correcting for multiple comparisons (SnPM using 5,000 randomizations, one-tailed threshold).

Paired analyses (SnPM) of the contrasts reported above for auditory hits relative to misses during Wii skateboarding levels 1 to 6 (high workload) were compared with the same contrasts for the transition period between Wii skateboarding levels (low workload) to assess the contribution of both workload and auditory task performance as underlying factors of inattentional deafness for alpha-band spectral power. No significant differential activity was found for any of the contrasts or for any of the ROI analyses in the left and right auditory cortex.

## Discussion

The aim of this study was to investigate whether EEG could reveal brain oscillations in the gamma and alpha frequency range, both pre- and post-stimulus presentation, that are indicative of inattentional deafness. The experiment's findings revealed that significant differences in brain activity between misses and hits (during the auditory stimulus difference detection task) occurred under the high workload skateboarding dual-task condition (presumably due to inattentional deafness) in some of the contrasts analyzed pre- and post-stimulus onset for the gamma and alpha frequency bands. Individual participant performance on the auditory stimulus difference detection task was also found to be correlated with differential brain activity for hits relative to misses on the auditory task. Furthermore, individual participant performance on the Wii skateboarding task was also found to be correlated with differential brain activity for hits relative to misses on the auditory task. Together, the results of this experiment investigating neural correlates of inattentional deafness are in accordance with the role of gamma- and alpha-band brain oscillations for attention and performance (Clayton et al., 2015).

To ensure that these results are a product of attentional processes thought to underlying inattentional deafness, a comparison of the relationship between auditory task hits and misses on the high workload dual-task condition occurring during levels 1 to 6 of the Wii skateboarding task was compared to a lower workload condition occurring during the transition period between Wii skateboarding levels. While there is some overlap in this differential workload-based analyses and the original high workload analyses in the gamma frequency range (Figures 3, 4 and Tables 1, 2), no significant activity was found for the differential workload-based analyses for the alpha frequency range. Even though this is the case, we still maintain that the results in the high workload dual-task condition are indeed a product of attentional processes thought to underlying inattentional deafness. Indeed, this is especially true for pre-stimulus activity in which it is difficult to imagine what other process than that of attention could account for prediction of future perceptual performance. We discuss limitations of the low workload condition below and why the results of the high workload condition alone do not overlap more with the contrast of high workload relative to the low workload condition.

While previous EEG and MEG studies have not found neural correlates of inattentional deafness/blindness in the gamma frequency range (Pitts et al., 2014; Hutchinson, 2019) our study, which used a more ecologically valid task, revealed gamma-band activity consistent with the attentional and performance-related functions previously reported in the literature (see Rieder et al., 2011 and Clayton et al., 2015, for reviews). For the contrast of auditory misses > hits for gamma-band activity averaged from −1000 to −50 msec pre-stimulus onset, significant differential activity was present over a single cluster spreading across the SFG, MFG, CG, and the SMFC (including pre-SMA) (see Figure 3A and Table 1). The location of this differential brain activity is consistent with many previous fMRI studies that have identified activity related to conditions indicative of an attentional bottleneck under high workload conditions (attentional overload) (Dux et al., 2006; Tombu et al., 2011; Szameitat et al., 2016). Of particular relevance is the fMRI study conducted by Durantin et al. (2017), which investigated auditory alarm perception in the context of a flight simulation piloting task and identified activity in the same brain region associated with episodes of inattentional deafness, implicating its involvement in processes of selective and divided attention. It has been suggested that these regions in the prefrontal cortex are involved with monitoring and evaluation of performance according to current task goals such that the attention system can continuously excite task-relevant processes and inhibit task-irrelevant processes (Clayton et al., 2015). Greater activation of this region under high workload conditions may reflect a greater need to inhibit task-irrelevant processes. In our case, in which participants are performing virtual skateboarding as the primary task, this would be auditory processes related to the secondary task.

The significantly greater level of differential alpha activity for auditory misses over hits in right hemisphere auditory processing brain regions both pre- (Figure 5A and Table 3) and post-stimulus (Figure 5B and Table 3) onset is consistent with the role of alpha oscillations as inhibiting task-irrelevant processes (Clayton et al., 2015). In the case of this experiment, the auditory task is considered the secondary task to be inhibited when the action demands of the primary task of virtual skateboarding are high.

Given the established role of cortical oscillations in attentional processes (Clayton et al., 2015), it was hypothesized that successful dual-task performance on the auditory task, indicated by an auditory hit, would elicit greater gamma-band activity in the relevant auditory processing regions of the brain. However, contrary to this prediction there was no significant differential gamma-band activity for pre- or post-stimulus contrasts of auditory hits greater than misses for this study. An additional way to investigate performance-related gamma activity is by performing a correlation analysis between overall individual auditory task performance and gamma-band brain activity for the contrast of auditory task hits relative to misses. The results for these correlation analyses for both pre- (Figure 3B and Table 1) and post-stimulus (Figure 3C and Table 1) analyses show significant gamma-band activity in left hemisphere brain regions involved with auditory processing including the primary auditory cortex and superior temporal gyrus/sulcus. This was especially the case for post-stimulus onset gamma-band performance correlated activity in which the focal point was localized to the left primary auditory cortex (Figure 3C and Table 1). However, for the pre-stimulus onset gamma-band performance correlated activity, the focal point was localized to the left inferior parietal cortex, including the temporal parietal junction (Figure 3B and Table 1). This result is interesting in that the IPL and TPJ are part of the ventral attention network VAN that is thought to be involved with processing of saliency (for a review see Vossel et al., 2014; Dehais et al., 2019a,b). However, in this case, the activity in these VAN brain regions is prior to the onset of the stimulus upon which saliency occurs. Given that there is no stimulus present for which saliency of the VAN to respond to, it may be the case that the VAN is being primed to respond to the presentation of a future stimulus. This is consistent with a previous finding (Marois et al., 2004; Todd et al., 2005) reporting that a demanding task can suppress TPJ activity and in return prevent the processing of incoming stimuli. It could also be the case that pre-stimulus onset gamma-band performance correlated activity centered in the IPL may correspond to processes related to auditory maintenance utilizing verbal rehearsal (Henson et al., 2000).

A correlation analysis between overall individual auditory task performance and alpha-band brain activity for the contrast of auditory task hits relative to misses was also conducted. No significant correlation with performance was present pre-stimulus onset with brain activity. However, post-stimulus brain activity (for the contrast of auditory hits relative to misses) did show a significant positive correlation with individual auditory task performance centered in the SMG region of the IPL and extending into the post- and precentral gyrus as well as the premotor cortex (Figure 5C and Table 3). One of the functions that the IPL is thought to take part in is acting as the somatosensory association cortex involved with perception of limb and body location in space (Callan et al., 2012; Limanowski and Blankenburg, 2016; Ruotolo et al., 2019). In accordance with theories of the roles of neural oscillations (Clayton et al., 2015), the greater alpha activity in the IPL may be associated with greater inhibition of task-related processes that are involved with representation of body control important for the virtual skateboarding task. The results are consistent with the hypothesis that brain regions important for executing the virtual skateboarding task are inhibited to a greater extent for individuals that do better on the auditory task. It should be noted, however, that there was no negative correlation between individual auditory task performance and virtual skateboarding task performance.

To investigate potential ongoing changes in gamma- and alpha-band activity as a result of stimulus presentation, an event-related spectral perturbation (ERSP) analysis was conducted. This analysis takes into account baseline gamma (alpha) band activity prior to stimulus onset relative to gamma (alpha) band activity after stimulus onset. The results of the ERSP analyses revealed significantly greater gamma-band activity for auditory hits relative to misses in the SFG and DLPFC (Figure 3D and Table 1) (No significant ERSP was found for the alpha band). These brain regions (SFG and DLPFC) within the inferior frontal junction have been implicated in the integration of the dorsal attention network (DAN involved with executive processing) and the VAN (involved with saliency of sensory stimuli) during task switches and/or attention shifts (Tamber-Rosenau et al., 2018). Activity in this region, in our study, is consistent with successful dual-task performance in which integration and switching between the DAN and VAN in response to an auditory stimulus would enhance auditory task performance during the concurrent virtual skateboarding task.

Although the event-related spectral perturbation (ERSP) analysis did not reveal any significant differential gamma-band activity in the auditory processing regions for hits relative to misses (when correcting for multiple comparisons), a subsequent region of interest (ROI) analysis conducted in the left auditory cortex showed a significant negative correlation between participants' performance in the skateboarding game and the brain activity associated with auditory hits compared to misses (Figure 3E). The findings align with Clayton et al. (2015) proposition that oscillations play both excitatory and inhibitory roles. Specifically, individuals who exhibit higher levels of selective attention toward the skateboarding task demonstrate reduced facilitatory gamma-band enhancement in the auditory processing regions when performing the auditory task. It should be pointed out that although auditory brain activity is negatively correlated with skateboard performance, there is no behavioral correlation between skateboard performance and the auditory task. One potential explanation for the lack of a negative correlation in behavior between the two tasks could be that the auditory task was not sensitive enough to detect threshold differences resulting from reduction in gamma-band facilitation. In this experiment, the auditory stimuli were presented at a level that was deemed to be loud but comfortably tolerable for the 30- to 40-min duration of the experiment. Therefore, the stimuli were always at a level that was quite audible. Additionally, it is possible that for individuals that are really good at the virtual skateboard game, they may be utilizing more automatic processing typical of expert skill that is somewhat independent from executive attentional processes which may therefore allow for these individuals to do relatively better at both the skateboarding and the auditory tasks. Further research is needed to explore the hypothesis of the interaction of controlled and automated processes and the effects of attention on multi-task performance.

One interesting finding in our study was the apparent lateralization of gamma-band activity to the left auditory cortex and alpha-band activity to the right auditory cortex. One potential reason for the left hemisphere auditory processing region laterality for gamma-band activity is related to the facilitation of specific acoustic features for perception. According to Ivry and Robertson (1998), the left hemisphere is specialized for processing high frequency patterns, e.g., the transitions in speech. The 100 msec duration chirp sounds with a frequency sweep from 2 to 4 kHz is characteristic of transitions found in speech sounds. Alternatively, it is also possible that this activity may represent verbal short-term memory rehearsal that is known to have a left hemisphere dominance (Henson et al., 2000). While post-stimulus gamma-band activity was lateralized to the left hemisphere auditory processing areas (Figure 3C and Table 1), post-stimulus alpha-band activity was lateralized to right hemisphere auditory processing areas (Figure 5B and Table 3). Alpha-band activity is thought to be related to inhibition of task-irrelevant processes (Clayton et al., 2015). Therefore, if the primary task is skateboarding, one would expect greater inhibition of brain activity in brain regions not involved with this primary task which include general auditory detection (“awareness”) in this case localized to the right hemisphere. Consistent with this hypothesis, in our study we see greater alpha-band activity for misses over hits in right hemisphere auditory brain regions signifying greater inhibition. Gamma reflects successful dual-task performance that varies across individuals and is facilitative (for the auditory task) in nature whereas alpha reflects the ability to suppress task-irrelevant processes (auditory) that may interfere with the focus of attention on the primary skateboarding task and is present across participants.

There are many potential reasons why we were able to find gamma-band activity in this study investigating inattentional deafness that were not present in other studies investigating inattentional deafness/blindness. (1) Most of basic MEG, fMRI, and EEG studies fail to induce high rate of inattentional deafness as they mainly report the effect of task workload on auditory processing (it is necessary to investigate misses as they are the potential occurrence of inattentional deafness/blindness). (2) Our study uses a challenging real-world immersive task that is likely to require greater engagement of the attention system, that in part utilizes gamma oscillations for facilitation of performance. (3) In many studies investigating inattentional deafness/blindness, there is only a single task upon which the participants are to act on and there is no task upon which the unexpected stimulus is acted on. In this case, it is primarily the saliency VAN that may be dominating the presence or absence of inattentional deafness/blindness. However, in our study, a dual-task paradigm was used; therefore, presence or absence of inattentional deafness relies on shifting of attention and successful divided attention in which both the VAN and the DAN are involved, thus utilizing greater gamma-band activity. (4) While most studies employing a dual secondary task utilize a very simple detection paradigm, we utilized an auditory task similar to that of a 1-back task in which the echoic memory of the previous stimulus needs to be maintained, thus engaging additional attention and related gamma-band activity. (5) Many previous studies simply did not do analyses to investigate source-localized gamma-band activity. (6) Our choice of the preprocessing pipeline to remove artifacts and extract brain activity and to conduct statistical analysis computed at the source level on the cortex may be more conducive to finding gamma-band activity differences between conditions. (7) Both of our tasks are continuous, thus requiring maintained dual-task attentional processes that may not be as prevalent when the tasks are more discrete in time. These are only some of the potential reasons why we were able to find gamma-band activity related to inattentional deafness/blindness where previous studies have not (Pitts et al., 2014; Hutchinson, 2019), even though it is known that gamma-band activity is important for attention and performance (Rieder et al., 2011; Clayton et al., 2015). It is beyond the scope of this study to determine which of these potentially multiple reasons are responsible for our finding of auditory performance-related gamma-band activity. Although we surmise that engaging the natural human attention system using complex ecologically valid continuous immersive tasks may have something to do with it.

It is important to acknowledge that there are a number of limitations to this study. The first involves the nature of the auditory difference detection task employed. Although the auditory difference detection task involves greater involvement of attention, which was the motivation for its use, one cannot discriminate whether performance is a result of missing the previous stimulus (prime) or the target, or both. Given the relatively low false alarm rate we can be somewhat confident that hits actually represent true hits and not guesses, it is difficult to ascertain whether a miss was due to an inability to sustain the previous stimulus in echoic memory, or an inability to perceive the current stimulus. Furthermore, we cannot discern whether misses were actually perceived, but there was an inability to be able to respond.

Another limitation is related to differences in the way inattentional deafness/blindness is defined by some as an unexpected event or object. In our task, as well as the tasks of many other studies investigating inattentional deafness, the participant is well aware that there are other stimuli not related to the primary task that are being presented as part of the experiment. In our study, since a dual-task paradigm is employed, the participants have explicit knowledge of the stimuli presented in the secondary task. Our experiment employed virtual skateboarding as the primary task and auditory difference detection as the secondary task. According to our definition of inattentional deafness/blindness, it is the process of selective attention being directed elsewhere that causes a lack of the ability to consciously perceive and act upon an object or event, that is of interest.

Although we utilized a comparison between the high workload condition (dual task during Wii skateboarding) and a low workload condition (transition period between Wii skateboarding levels) to assess the involvement of attentional load on performance above that of just perceptual processing, the results were not conclusive perhaps due to limitations of the low workload condition. Even during the low workload condition, performance on the auditory task was considerably low (hit rate equal to 0.431), suggesting that it may not actually be a low workload condition but rather also a moderately high workload condition that is significantly easier than the high workload condition occurring while doing Wii skateboarding (hit rate equal to 0.189). While the transition period between Wii levels certainly appears to have less dual-task attentional load, this period nevertheless involves a button press to continue to the next level that may direct attention away from the auditory task. The additional activity in IFG known to be involved with stimulus induced attentional switching (Doeller et al., 2003; Perianez et al., 2004; Tamber-Rosenau et al., 2018) in the high workload vs. low workload comparison that are not present in the high workload contrast alone (ERSP analyses Figures 3, 4 and Tables 1, 2), may reflect to some extent these differences in task-related attentional switching demands. Another potential reason why the low workload condition did not have the expected high performance as predicted, may be due to the continuous nature of the tasks. It may be the case that the suppressive attentional mechanisms active during the high workload dual-task condition (during Wii skateboarding) are maintained during the brief transition period between levels.

The apparent low auditory performance level for both the high workload and the low workload conditions represents a potential floor effect that could have important implications with respect to the results found and lack of results predicted to be found in this study. When performance is so low, it is difficult to discern whether participants were actually doing the auditory task or not. The degree of dual-task attentional engagement is considerably different in these two cases. Given the significant differential activity between misses and hits in theoretically relevant regions involved with attentional processing and inattentional deafness (Durantin et al., 2017), we do not believe this to be the case in our study. In addition, the variability in auditory task performance across participants was enough to reveal significant correlation with brain activity for hits relative to misses in theoretically relevant brain regions (mainly the auditory processing regions, see Figures 3B, C and Table 1). However, as discussed above (with respect to the high workload vs. low workload comparison), the apparent floor effect in performance may be responsible for the lack of findings that were predicted. It was predicted that there would be a tradeoff between Wii game performance and auditory task performance. However, this relationship was not found at the individual participant level across the course of the Wii games composing the experiment or across participants (See behavioral results). Although no significant correlation was found between the participants' performance in the Wii game and their performance in the auditory task, an interesting discovery was made. There was a significant negative correlation between the maximum Wii score achieved by the participants and the gamma-band ERSP activity in the left auditory cortex (see Figures 3E, 4D). This suggests that individuals who are more proficient in the Wii game exhibit lower neural processing of auditory stimuli in this particular brain region. This could represent suppressive processes resulting from inattentional deafness and directed attention to the Wii skateboarding task over the auditory task. The floor effect in auditory task performance may be hiding the behavioral relationship to support this conclusion.

Another potential limitation of our study is that the artifact removal and brain activity extraction procedures utilizing regression of head movement correlated activity, such as ASR, ICA, and IC label, are not able to completely separate artifact from brain activity. It is likely that some relevant task-related activity has been removed from the data along with the artifacts. Therefore, the lack of finding of a difference in brain activity between conditions does not mean that it is not actually present. It is also possible that differential activity found to be present between conditions is the result of differing degree of artifacts. A major concern when reporting gamma-band activity differences for EEG and MEG is the correspondence in the same frequency region as that of muscle activity (Yuval-Greenberg et al., 2008; Muthukumaraswamy, 2013). Muscle activity is in the range from approximately 20 to 300 Hz and can be picked up by EEG electrodes especially for muscles involved with eye movement including saccades as well as muscles on the face, neck, and shoulders (Yuval-Greenberg et al., 2008; Muthukumaraswamy, 2013). Given the high movement demands and the visual motor nature of the Wii skateboarding task, these muscle-related artifacts are of considerable concern. As pointed out by Muthukumaraswamy (2013), one way to address potential muscle artifacts in EEG is to remove them using ICA (a procedure used in our preprocessing pipeline). One limitation Muthukumaraswamy (2013) points out of using ICA to remove muscle artifacts is that it requires an expert to identify which components correspond to brain and artifact components. The development of ICLabel (Pion-Tonachini et al., 2019), that was used in our study, which is known to have expert level performance at selection of brain and artifact components, to some degree accounts for this apparent limitation. While it is known that movement artifacts related to eye movement and muscle activity can contaminate EEG data, we took several steps to attempt to remove potential muscle-related artifacts from the EEG data. The primary method used was ICA combined with ICLabel that identifies whether a component is mostly brain, muscle, eye movement, and other artifacts. All subjects had components for eye movement and other muscle-related artifacts that were removed from the data. In addition, ASR was used to remove non-stationary artifacts that may arise from movement. Nevertheless, it is possible that there is greater muscle activity during misses than hits as a result of greater involvement in the Wii skateboarding game during these time periods that could confound our results. One reason we do not believe this to be the case is that the muscle activity for eye movement and saccades should be source-localized to regions on the edges of the brain in the orbital frontal cortex near the eyes or in the inferior temporal cortical regions near the neck muscles (this was not the case, see Figures 3–5). The localization of brain activity in our study in theoretically relevant regions does suggest that they are not merely the result of contamination by muscle artifact. Another potential confound is the presence of a button press for hits but not for misses. While this is not an issue for pre-stimulus contrasts and those involving correlation across participants, it may be so for post-stimulus contrasts including the ERSP analysis. Given the lack of brain activity in motor planning or execution related areas involved with pressing a button, we do not believe that this was a confounding issue for our results.

## Conclusion

The results of our experiment suggest some of the potential underlying neural attentional processes that are responsible for inattentional deafness. Under conditions of high workload, brain regions in the prefrontal cortex (SMFC and pre-SMA) are engaged to a greater extent as a result of greater processing related to monitoring and evaluation of which task-relevant brain processes to facilitate and which task-irrelevant brain processes to inhibit dependent on action demands. The result of this process is selective inhibition of task-irrelevant processes reflected by greater alpha activity (inhibition) in auditory processing regions evident for misses over hits. Additionally, in some individuals, efficient dual-task performance is facilitated by excitation of task-relevant processes in auditory processing regions reflected by greater gamma-band activity for hits over misses correlated with overall participant level auditory task performance. Gamma brain activity in regions in the inferior frontal junction (e.g., IFG and DLPFC) is important in integrating DAN (executive) and VAN (salience) during task switches and attention shifts in multi-task situations reflected by greater ERSP activity for hits over misses in our study. Although our study reveals some of the potential processes involved with occurrence of inattentional deafness, further research is needed using different tasks that better modulate workload and avoid a potential floor effect to see how well they generalize. Additionally, further experiments investigating the functional connectivity between frontal and perceptual processing areas are needed to better understand the mechanisms involved with selective excitation of task-relevant processes and selective inhibition of task-irrelevant processes as well as the processes involved with attentional shifts during multi-task situations.

## Data availability statement

The raw data supporting the conclusions of this article will be made available by the authors, without undue reservation.

## Ethics statement

The studies involving human participants were reviewed and approved by Advanced Telecommunications Research Institute International Human Subject Review Committee. The patients/participants provided their written informed consent to participate in this study. Written informed consent was obtained from the individual(s) for the publication of any potentially identifiable images or data included in this article.

## Author contributions

DC: conceptualization, investigation, methodology, software, formal analysis, visualization, and writing—original draft. TF: investigation. FD: writing—original draft and writing—review and editing. SI: writing—review and editing and funding acquisition. All authors contributed to the article and approved the submitted version.
